# Hospitalizations and cardiac sarcoidosis: insights into presentation and diagnosis from the nationwide readmission database

**DOI:** 10.3389/fcvm.2024.1475181

**Published:** 2024-11-19

**Authors:** Jacob Abraham, Kateri Spinelli, Hsin-Fang Li, Tuan Pham, Mansen Wang, Farooq H. Sheikh

**Affiliations:** ^1^ Center for Cardiovascular Analytics, Research and Data Science (CARDS), Providence Heart Institute, Providence Research Network, Portland, Oregon; ^2^ Adventist Health, Portland, Oregon; ^3^ Advanced Heart Failure Program, MedStar Washington Hospital Center, Georgetown University School of Medicine, Washington, DC, United States

**Keywords:** cardiac sarcoidosis, national readmission database, ventricular arrhythmias, heart failure, conduction disorders

## Abstract

**Introduction:**

Cardiac sarcoidosis (CS) is an increasingly recognized cause of cardiac disease. Because the clinical presentation of CS is non-specific, the diagnosis is often delayed. Early detection is essential to initiate treatments that reduce the risk of heart failure (HF) and arrhythmic death. We therefore aimed to describe the features of CS hospitalizations during which the initial diagnosis of CS is made.

**Methods:**

We performed a retrospective analysis of hospitalizations from 2016 to 2019 in the Nationwide Readmission Database (NRD). Hospitalizations with a primary diagnosis suggestive of CS (HF/cardiomyopathy, cardiac arrest, arrhythmias, or heart block) were categorized into cases with and without CS as a secondary diagnosis (CS+ and CS−, respectively). One-to-one propensity score matching (PSM) was performed.

**Results:**

The CS+ cohort comprised 1,146 hospitalizations and the CS− cohort 3,250,696 hospitalizations. The CS+ cohort included patients who were younger and more often male. PSM resulted in highly matched cohorts (absolute standardized mean difference <0.1). Primary diagnoses of ventricular arrhythmias (VA) or heart block were more frequent in matched CS+ hospitalizations, whereas primary diagnosis of HF/cardiomyopathy was more frequent in matched CS− hospitalizations. The matched CS+ group exhibited higher rates of in-hospital procedures and longer length of stay. In-hospital mortality and 30-day readmission were similar between matched cohorts.

**Discussion:**

These findings highlight increased rates of CS in younger males with primary diagnoses of VA and heart block, and increased use of diagnostic and therapeutic interventions such as pacemaker and left ventricular assist device implantation, and could aid clinicians in more timely diagnosis and treatment of CS.

## Introduction

Sarcoidosis is a multi-system granulomatous disease of uncertain etiology, variable population prevalence, and protean clinical manifestations (1). Cardiac sarcoidosis (CS) can present as sudden cardiac death (SCD), heart failure (HF), arrhythmias, and/or conduction disorders (1, 2). The prevalence of symptomatic cardiac involvement has been historically reported as 5%, though asymptomatic cardiac involvement may affect 20%–25% of patients with pulmonary or systemic sarcoidosis (1, 3). With use of contemporary multi-modality cardiac imaging and updated diagnostic guidelines, however, rates of hospitalization for sarcoidosis have increased (4–8) Immunosuppressive and novel non-steroidal treatment approaches can potentially delay the progression of cardiac sarcoidosis, underscoring the need for early diagnosis and treatment (1, 9).

CS is diagnosed when cardiac symptoms develop in a patient with a diagnosis of systemic sarcoidosis or when HF, SCD, or arrhythmias of unclear etiology are ultimately attributed to granulomatous inflammation (7, 8). The manifestations of CS are non-specific and overlap with other disorders (10–12). For this reason, a high index of suspicion for CS is needed to diagnose CS. To date, there is limited data assessing differences in the primary clinical presentation of CS from non-CS cardiac disorders that could aid clinicians in the diagnostic process (13). We hypothesized that differences in clinical presentation, demographics, testing, and outcomes can be identified between hospitalizations with vs. without a secondary diagnosis of CS. Therefore, we compared hospitalizations for primary diagnoses suggestive of CS between cases with and without a CS secondary diagnosis in a national readmissions database.

## Methods

### Population and data source

We retrospectively analyzed hospitalizations in the Healthcare Cost and Utilization Projection (HCUP) Nationwide Readmissions Database (NRD) from 2016 through 2019 (14). This database is a United States (US) all-payer administrative dataset constructed from the individual state inpatient hospitalization databases, developed and made available by the US Agency for Healthcare Research and Quality (AHRQ). The NRD is a de-identified dataset; hospitalizations (inpatient admissions) are the units of measurement. For each admission, the NRD includes hospital characteristics, demographics, comorbidities, primary and secondary diagnosis codes, procedures performed, in-hospital outcomes [length of stay (LOS), mortality], and total charges. Clinical information prior to admission, medication data, and clinical information not coded by ICD codes during the in-hospital stay are not included in the NRD. If a patient readmitted within the calendar year, a unique identifier is added to the NRD record to allow for tracking between the index admission and subsequent readmissions within the calendar year. The study interval was chosen to align with the introduction of ICD-10 codes in October 2015. At the time of data analysis, 2019 was the most recent year of the NRD dataset that was available from HCUP. We chose to include data through the end of 2019 to avoid potential confounding effects of the COVID-19 pandemic in 2020 and later.

The study cohort, comorbidities, and procedures were identified using International Classification of Diseases, 10th Revision, Clinical Modification codes (ICD-10-CM) and Procedure codes (ICD-10-PCS) (Supplementary Table S2). At least two sources were cross-referenced for each code category; sources included published medical literature, HCUP clinical classification software refined, and internal programmatic quality and operations codes list.

**Figure 1:**
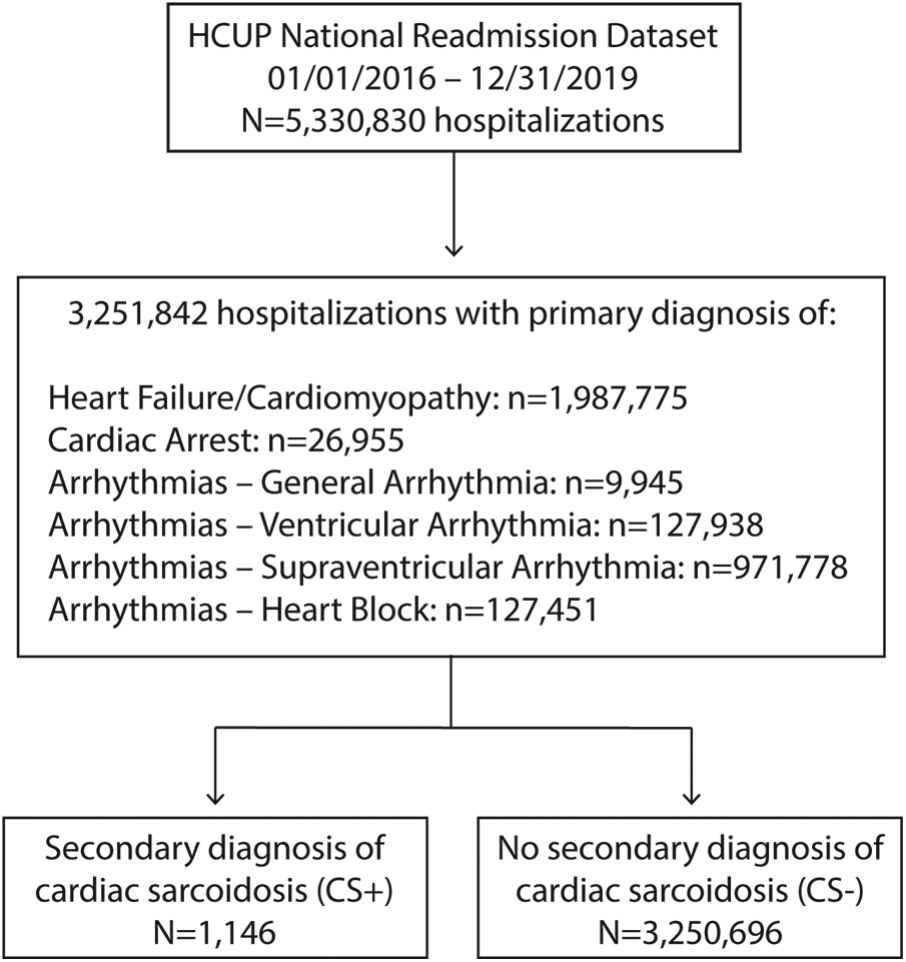
Study selection flow chart. CS, cardiac sarcoidosis; HCUP, health care utilization project.

The study cohort consisted of hospitalizations with a primary discharge diagnosis of HF/cardiomyopathy, cardiac arrest, general arrhythmia, ventricular arrhythmia (VA), supraventricular arrhythmia, or heart block in the first visit within the calendar year (Figure 1). Hospitalizations with a primary diagnosis of CS were excluded from the analysis cohort. For the 30-day readmission outcome, we excluded cases from December to avoid lack of 30-day follow-up, since cases cannot be tracked across years in the NRD. This study was approved by the Providence St. Joseph Health institutional review board, with waiver of informed consent.

### Study aims

The primary aim was to compare demographics and clinical characteristics of patients hospitalized with a primary diagnosis suggestive of CS who also had a secondary diagnosis of CS (CS+) vs. those who do not (CS−). As outlined above, the study dataset included hospitalizations with one of the six primary diagnoses suggestive of CS during the study time frame. Those hospitalizations were then sub-grouped into the presence (CS+) or absence (CS−) of cardiac sarcoidosis (ICD-10: D86.85) as a secondary diagnosis. The secondary aims were to (1) compare in-hospital procedures, outcomes, and 30-day readmissions between subgroups, and (2) to examine temporal trends in admissions, in-hospital mortality, and 30-day readmissions between the subgroups.

### Statistical analysis

Demographics, in-hospital procedures, 30-day readmissions, and outcomes within the calendar year were summarized for each group. Length of stay was reported as median and interquartile range (IQR) (25th, 75th percentile) based on non-normal distribution of the variable. All other continuous variables were summarized using descriptive statistics (*n*, mean ± SD). Categorical variables were summarized using frequencies and percentages. Prevalence estimates were weighted using survey analysis methods with “DISCWT” as the weight variable, “HOSP_NRD” as the clustering variable, and accounting for the different strata in the NRD design using the “NRD_STRATUM”, following AHRQ recommendations. This approach aimed to ensure accurate national representative estimates for the US hospitalizations (14).

To balance potential confounding factors between CS+ and CS− cases, a propensity score matching (PSM) method was implemented. Multivariable logistic regression was used to calculate propensity scores and estimate the probability of cardiac sarcoidosis diagnosis based on age, sex, year of admission, comorbidities listed in Table 1, urban/rural hospital type, and teaching/non-teaching hospitals. A CS+ case was matched with a CS− case using the greedy nearest-neighbor matching algorithm without replacement. The caliper was set at 0.25. Covariate balance between cardiac sarcoidosis and non-cardiac sarcoidosis was assessed using standardized mean differences (SMD).

**Table 1:** Demographics.

Variable^a^	CS+ (*N* = 1,146)	CS−			
		Unmatched (*N* = 3,250,696)	SMD	Matched (*N* = 1,146)	SMD
Age, years, median (IQR)	57 (49, 65)	73 (62, 83)	1.108	57 (49, 65)	0
Sex, female	458 (40%)	1,546,524 (48%)	0.154	458 (40%)	0
Comorbidities					
Cerebrovascular disease	15 (1%)	82,482 (3%)	0.09	12 (1%)	−0.003
Diabetes without chronic complications	148 (13%)	470,169 (14%)	0.045	152 (13%)	0.004
Diabetes with chronic complications	223 (19%)	843,617 (26%)	0.155	220 (19%)	−0.003
Hypertension, complicated	307 (27%)	706,749 (22%)	−0.118	308 (27%)	0.001
Hypertension, uncomplicated	211 (18%)	778,340 (24%)	0.136	211 (18%)	0
Chronic pulmonary disease	255 (22%)	1,026,731 (32%)	0.212	246 (21%)	−0.008
Obesity	321 (28%)	766,189 (24%)	−0.102	318 (28%)	−0.003
Peripheral vascular disease	39 (3%)	325,376 (10%)	0.266	37 (3%)	−0.002
Renal failure, severe	16 (1%)	148,996 (5%)	0.188	20 (2%)	0.004
Valvular disease	20 (2%)	159,959 (5%)	0.178	20 (2%)	0
Hospital type, teaching/non-teaching					
Metropolitan non-teaching	85 (7%)	816,425 (25%)	0.494	84 (7%)	−0.001
Metropolitan teaching	1,035 (91%)	2,154,287 (66%)	−0.61	1,038 (91%)	0.003
Non-metropolitan hospital	26 (2%)	279,984 (9%)	0.282	24 (2%)	−0.002
Hospital type, urban/rural					
Large metropolitan areas ≥1 million residents	894 (78%)	1,798,121 (55%)	−0.496	890 (78%)	−0.004
Small metropolitan areas <1 million residents	226 (20%)	1,172,591 (36%)	0.371	232 (20%)	0.005
Micropolitan areas	22 (2%)	211,323 (7%)	0.23	19 (2%)	−0.003
Not metropolitan or micropolitan (non-urban residual)	4 (0.4%)	68,661 (2%)	0.16	5 (0.4%)	0.001
Median household income					
<$45,250	270 (24%)	935,141 (29%)		359 (32%)	
$45,250–$57,500	252 (22%)	862,140 (27%)		288 (25%)	
$57,500–$76,500	266 (24%)	786,529 (25%)		275 (24%)	
$76,500+	336 (30%)	622,694 (19%)		213 (19%)	

CS, cardiac sarcoidosis; SMD, standardized mean difference.

Data presented as *n* (%) unless otherwise noted.

^a^ All variables in Table 1 except median household income were included in the propensity score model. Year of admission was also included in the propensity score model.

Comparisons of the outcomes were performed after PSM. Categorical variables were compared with McNemar tests. Continuous variables were described using paired *t*-tests. Medians were compared using Wilcoxon's rank sum test. Missing data rates were <1% for all variables except median household income (1.4%). Significance was considered at *p*-values <0.05. All analyses were conducted using SAS version 9.4 (SAS Institute Inc). Figures were designed and edited in SAS, GraphPad Prism 9, and Adobe Illustrator 2022.

## Results

**Figure 2:**
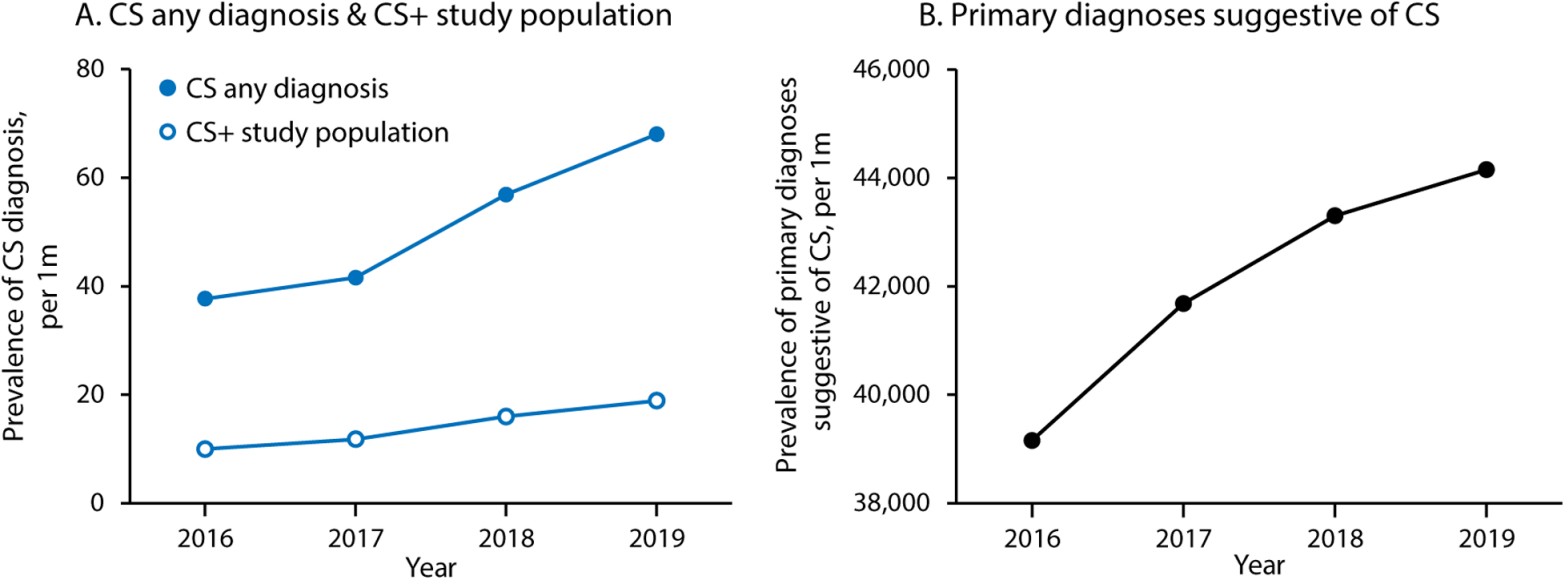
Trends in cardiac sarcoidosis and related diagnoses over time. **(A)** Shows prevalence of CS as any diagnosis (primary or secondary) and within the study population (cases with one of the six primary diagnoses suggestive of CS and a secondary diagnosis of CS). **(B)** Shows prevalence of the six primary diagnoses suggestive of CS (heart failure/cardiomyopathy, cardiac arrest, general arrhythmia, ventricular arrhythmia, supraventricular arrhythmia, and heart block). CS, cardiac sarcoidosis.

Between 2016 and 2019, there were 1,146 hospitalizations with a primary diagnosis suggestive of CS plus secondary diagnosis of CS (CS+) and 3,250,696 hospitalizations in patients with a primary diagnosis suggestive of CS without a secondary diagnosis of CS (CS−) (Figure 1). The CS+ population increased modestly each year over the study period (Figure 2). Over these same four years, CS as any diagnosis increased over time, from 37.7 per million in 2016 to 68.0 per million in 2019. The six primary diagnoses suggestive of CS also increased over the study period, from 39,155 per million in 2016 to 44,155 per million in 2019.

The CS+ cohort included patients who were younger (57 vs. 73 years old) and more likely male (60% vs. 52%) (Table 1). Cases without CS had higher rates of nearly all comorbid conditions, including hypertension, diabetes mellitus, obesity, valvular disease, peripheral vascular disease (PVD), and renal dysfunction (Table 1). Based on All Patients Refined Diagnosis Related Groups (APR-DRG) risk of mortality, CS+ cases had a higher likelihood of dying (major and extreme likelihood 55% vs. 50%) (Supplementary Table S1). CS+ hospitalizations occurred more often at a metropolitan teaching hospital (91% for the CS+ cohort vs. 66% for the CS− cohort).

PSM resulted in a well-matched CS population, with absolute standardized mean differences of less than 0.1 for all matching variables (Table 1; Supplementary Figure S1). After matching, demographics, comorbidities, and hospital characteristics were nearly equal between the groups (Table 1). Median household income was not included as a matching variable and remained higher in the CS+ group after matching ($76,500+, 30% vs. 19%, CS+ vs. CS−).

After matching, hospitalizations in the matched CS+ group more often presented with a primary diagnosis of VA (36% vs. 8%) or heart block (12% vs. 3%) (Central Illustration). Those in the matched CS- group more often presented with a primary diagnosis of HF/cardiomyopathy (52% vs. 42%) or supraventricular arrhythmia (36% vs. 10%).

**CENTRAL ILLUSTRATION:**
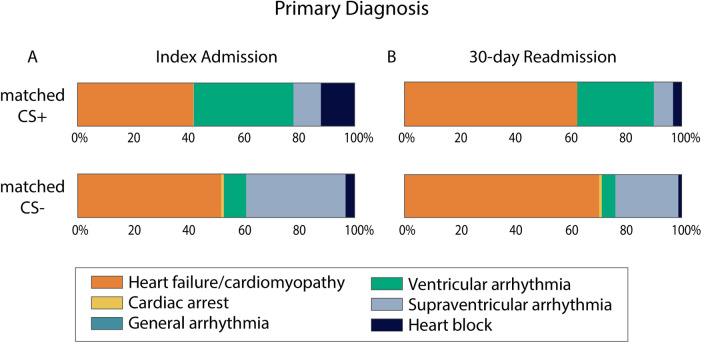
Primary diagnosis for admissions with and without secondary diagnosis of cardiac sarcoidosis. Percent of cases in the matched CS+ and matched CS− groups that presented with each of the six primary diagnoses that are suggestive of CS. Index admission cases are summarized in panel A. 30-day readmission cases are summarized in panel B. For 30-day readmissions, *N* = 162 for the matched CS+ group and *N* = 160 for the matched CS− group. CS, cardiac sarcoidosis.

In-hospital procedures were performed more frequently in the matched CS+ group compared to the matched CS- group, including right heart catheterization (RHC) (17% vs. 8%, *p* < .0001), endomyocardial biopsy (4% vs. 0.6%, *p* < .0001), ventricular assist device (VAD) implantation (2% vs. 0.5%, *p* = 0.004), and permanent pacemaker insertion (31% vs. 6%, *p* < .0001) (Table 2). Furthermore, the matched CS+ cohort had significantly longer length of stay (4 (2–8) days vs. 3 (2–6) days, *p* < .0001) (Table 3). Nevertheless, in-hospital mortality during the index admission remained similar between the matched CS+ and matched CS- cohorts (2% vs. 3%, *p* = 0.08). All-cause 30-day readmission did not differ between groups (14% vs. 14%, *p* = 0.9), nor did the number of readmissions over the calendar year (*p* = 0.5269). Over the study period, in-hospital mortality during index admission and 30-day readmission rates were variable and did not statistically differ between cohorts (Supplementary Figure S2).

**Table 2:** In-hospital procedures.

Variable	CS+ (*N* = 1,146)	CS−		CS+ vs. CS-matched *p*-value
		Unmatched (*N* = 3,250,696)	Matched (*N* = 1,146)	
Pacemaker defibrillator	350 (31%)	197,310 (6%)	68 (6%)	<.0001
VAD	21 (2%)	8,137 (0.3%)	6 (0.5%)	0.0037
tMCS	24 (2%)	10,809 (0.3%)	12 (1%)	0.0438
ECMO	6 (1%)	1,861 (0.1%)	0 (0%)	0.0142
Transplant	21 (2%)	3,139 (0.1%)	8 (1%)	0.0151
18F-PET	0 (0%)	3 (0%)	0 (0%)	N/A
Cardiac MRI	8 (0.7%)	672 (0.02%)	2 (0.2%)	0.0572
RHC	199 (17%)	118,255 (4%)	86 (8%)	<.0001
Coronary angiography	187 (16%)	274,443 (8%)	152 (13%)	0.0395
Endomyocardial biopsy	48 (4%)	331 (0.1%)	7 (0.6%)	<.0001

MRI, magnetic resonance imaging; CS, cardiac sarcoidosis; ECMO, extracorporeal membrane oxygenation; 18F-PET, fluorine 18 positron emission tomography; RHC, right heart catheterization; tMCS, temporary mechanical circulatory support; VAD, ventricular assist device.

Data presented as *n* (%) unless otherwise noted.

**Table 3:** Outcomes.

Variable	CS+ (*N* = 1,146)	CS−		CS+ vs. CS− matched *p*-value
		Unmatched (*N* = 3,250,696)	Matched (*N* = 1,146)	
LOS, median (IQR)	4 (2, 8)	3 (2, 6)	3 (2, 6)	<.0001
Index visit mortality	18 (2%)	94,398 (3%)	30 (3%)	0.0800
Discharge disposition				<.0001
Home or self-care	879 (77%)	1,937,961 (60%)	843 (74%)	
Transfer to Short-term Hospital	29 (2%)	35,415 (1%)	21 (2%)	
Transfer other: includes SNF, ICF, another type of facility	31 (3%)	479,050 (15%)	67 (6%)	
HHC	183 (16%)	659,303 (20%)	164 (14%)	
AMA	6 (1%)	42,951 (1%)	21 (2%)	
Died	18 (2%)	94,398 (3%)	30 (3%)	
30-day readmission (all-cause)	162 (14%)	512,557 (16%)	160 (14%)	0.9043
30-day readmission (HF/cardiomyopathy)	37 (3%)	160,875 (5%)	53 (5%)	0.0853
In-hospital mortality at 30-day readmission event	9 (6%)	35,428 (7%)	7 (4%)	0.6260
In-hospital mortality over the calendar year	51 (4%)	212,651 (7%)	53 (5%)	0.8409
Readmissions per calendar year				0.5269
0 readmission	715 (62%)	2,029,107 (62%)	748 (65%)	
1 readmission	234 (20%)	676,561 (21%)	225 (20%)	
2 readmissions	90 (8%)	280,180 (9%)	85 (7%)	
3 readmissions	49 (4%)	127,886 (4%)	37 (3%)	
>4 readmissions	58 (5%)	136,962 (4%)	51 (4%)	

AMA, against medical advice; HF, heart failure; HHC, home health care; ICF, intermediate care facility; IQR, interquartile range; LOS, length of stay; SNF, skilled nursing facility.

Data presented as *n* (%) unless otherwise noted.

## Discussion

In this analysis of the Nationwide Readmissions Database, we identified primary diagnoses suggestive of CS (cardiac arrest, HF, arrhythmia, conduction disease), then defined cohorts of hospitalizations based on presence (CS+) or absence (CS-) of CS as a secondary diagnosis and compared propensity matched CS+ and CS− cohorts. This analysis yields several important observations. First, the CS+ cohort was comprised of patients who were significantly younger, more often male, and had fewer cardiac and non-cardiac comorbidities (valvular disease, hypertension, chronic obstructive pulmonary disease, PVD, or severe renal failure). Second, hospitalizations for matched CS+ cases more frequently related to a primary diagnosis of ventricular arrhythmia or heart block, whereas hospitalizations in the matched CS- cohort had higher rates of HF. Third, the matched CS+ cohort had higher rates of invasive diagnostic procedures (endomyocardial biopsy, RHC, coronary angiography) and invasive therapies [implantation of pacemaker/defibrillator, left ventricular assist device (LVAD), or heart transplant]. Fourth, despite having a higher rate of major likelihood of dying, cases in the matched CS+ cohort had the same in-hospital mortality and all-cause and HF-specific readmission rates at 30 days.

In contrast to prior publications that analyzed administrative datasets beginning with a primary diagnosis of sarcoidosis, we defined the study population by selecting primary diagnoses that could represent manifestations of CS. The rationale for this analysis was to identify demographic and clinical features that may assist clinicians to identify CS patients amongst a population presenting with HF/cardiomyopathy, cardiac arrest, arrhythmias, or heart block (e.g., primary diagnosis suggestive of CS).

In taking this analytic approach, our study yields insights that could potentially refine clinical suspicion for CS when confronting patients with unexplained HF, cardiac arrest, or arrhythmias. In particular, we found a younger and predominantly male patient population with higher rate of presentations with VT or conduction block in the cohort with a secondary CS diagnosis. The results of this analysis have potential implications for diagnosing and treating CS. Specifically, our results indicate that CS should be strongly suspected in younger, male patients presenting with ventricular arrhythmias or conduction system disease and should prompt additional diagnostic testing, including fluorine-18 fluorodeoxyglucose-positron emission tomography/computed tomography (18F-FDG PET/CT) scans or cardiac magnetic resonance imaging (MRI). Patients suspected of having CS are more likely to require RHC and endomyocardial biopsy. Additionally, permanent pacemaker and durable LVAD are more frequently indicated in CS+ patients.

Our findings contrast with an earlier study of sarcoidosis hospitalizations by Patel et al. using the National Inpatient Sample from 2005 to 2014. Because of the earlier time frame, the authors identified sarcoidosis and cardiovascular manifestations using ICD-9 diagnosis codes, which are less extensive than ICD-10 codes (4). They reported a female predominance and heart failure and arrhythmias (not further specified) as the major cardiac causes of admission in sarcoidosis. The differences in findings between the current study and that of Patel et al. may be explained by the fact that Patel et al. defined their study population as any diagnosis of sarcoidosis, excluded ischemic heart disease hospitalizations, and used a different and a less contemporary administrative database. Additionally, in February 2017, the Japanese Circulation Society published new guidelines for the diagnosis and treatment of CS that elevated 18F-FDG PET/CT tracer uptake in the myocardium or late gadolinium enhancement by MRI from minor to major diagnostic criteria (7). In August 2017, the Society of Nuclear Medicine and Molecular Imaging and the American Society of Nuclear Cardiology published a consensus document on the role of 18F-FDG PET/CT in cardiac sarcoid detection and therapy monitoring (15). These guideline statements may have increased the use of MRI or PET, which could increase the incidence of CS as has been observed in Finland, but we cannot draw any causal inferences from our analysis (16).

Due to the challenges of diagnosis and evolving diagnostic criteria, the true prevalence of isolated cardiac sarcoidosis is difficult to ascertain. In a retrospective study of 286 patients with suspected CS by Takaya et al, 7.3% of patients were diagnosed with isolated CS and 22% diagnosed with systemic CS utilizing updated Japanese Circulation Society guidelines. There were no demographic differences between patients with and without CS. In a secondary analysis of the ILLUMINATE-CS Japanese registry study, clinical characteristics and prognosis were compared between patients with isolated cardiac sarcoidosis and cardiac sarcoidosis with concomitant systemic disease. The primary outcome was a combined endpoint of all-cause death, hospitalization for heart failure, or fatal ventricular arrhythmia events. Among 475 patients with CS (mean age, 62.0 ± 10.9 years; female sex, 59%), 25.1% were diagnosed with isolated CS. Isolated CS patients had a higher prevalence of hospitalization for heart failure and lower left ventricular ejection fraction than those with systemic cardiac sarcoidosis. Isolated CS was a significant risk factor for the primary outcome in an unadjusted model, but this association was not maintained in a multivariable model.

The in-hospital mortality, 30-day readmission rates, in-hospital mortality over the calendar year, and readmissions per calendar year (Table 3) were similar amongst the unmatched study cohorts despite higher APR-DRG major mortality risk in the CS+ cohort. This finding warrants further investigation, though it may be related to the younger age and fewer non-cardiac comorbidities of the CS+ cohort, as well as the higher rate of admission to urban teaching hospitals. The lack of difference in mortality is particularly noteworthy given the fact that the CS+ cohort underwent invasive diagnostic and therapeutic procedures at a higher rate, including temporary mechanical circulatory support, ECMO, and heart replacement therapies (LVAD or transplant), suggesting a more severe clinical trajectory amongst a subset of CS+ patients. The observation that LOS was longer for CS+ patients compared to CS− patients is notable given the fact that CS+ patients were found to have a lower HF/cardiomyopathy incidence relative to CS− patients in this analysis. Longer LOS may also have been related to the higher rates of invasive procedures in the CS+ patients, which in turn could be related to the subsequent diagnosis of CS. Given the limitations of the current dataset, it is unclear what variables may have contributed to differences in LOS. This finding warrants further investigation and verification with additional datasets. It is possible that tertiary or quaternary diagnoses not captured in NRD may explain the LOS difference given the fact that sarcoidosis represents a systemic disease which places patients at risk for significant comorbidities. Additionally, delays in diagnosis or more severe disease presentation amongst CS+ patients may contribute to these observed differences.

We performed propensity score matching using covariates of age, sex, year, and clinically relevant comorbidities to minimize the effects of confounding. The very low SMD for all covariates indicates a highly balanced match that limits the effects of potential confounders. The major observations of the study remained significant after matching, strengthening confidence in our findings. The heightened risk of adverse outcomes for patients with CS has been previously studied in several national European registries. A Danish registry analysis demonstrated an increased lifetime hazard for HF, atrial/ventricular arrhythmias, heart block/need for pacemaker, and all-cause mortality in patients with systemic sarcoidosis (17). In a nationwide 18-year registry inclusive of 351 patients with CS in Finland, Ekstrom and colleagues identified atrioventricular block and VA as common presenting manifestations. Sudden death accounted for 80% of all deaths (18). Similarly, Takaya et al. showed that the rate of cardiac death of hospitalization for HF was higher in patients with isolated CS than systemic CS. Taken together with our finding that VA is more frequent in the CS+ cohort, these observations highlight the urgency of appropriate diagnostic testing and therapeutic interventions.

Finally, we report a rising prevalence of CS during the study period (37.7 per million in 2016 to 68.0 per million in 2019). This observation likely underestimates the actual disease prevalence as this analysis is limited to inpatients. A rising prevalence of CS in this North American registry corroborates recent observations from Europe (19). Though the reasons underlying this observation cannot be determined from the present analysis, it is reasonable to surmise that heightened awareness of CS amongst clinicians, coupled with increased use of advanced cardiac imaging (cardiac MRI and FDG-PET/CT scans), has contributed to higher rates of diagnosis. Clinically manifested CS represents a fraction of the total disease burden, underscoring the need for clinicians to seek out the diagnosis of CS in order to prevent significant morbidity and mortality (19).

## Limitations

There are limitations to this observational study. First, as with all administrative databases, the NRD is inherently subject to potential coding errors, in particular for procedures that are typically coded using current procedural termination (CPT) codes which are not available in the NRD, such as endomyocardial biopsy, cardiac MRI, and PET scans. In addition, the NRD does not include medication information or information prior to the index hospital admission, such as patients’ clinical treatment, procedures, or previous hospitalization data. Follow up studies using clinically adjudicated datasets that follow the clinical trajectory of CS patients would be useful. Second, the NRD excludes interstate hospitalizations and does not link admissions across calendar years, which could underestimate readmission rates. Third, we excluded cases with a primary diagnosis of CS to enrich for patients diagnosed during the index hospitalization. However, we are unable to determine with certainty the timing of an initial CS diagnosis nor were we able to ascertain how the diagnosis of CS was made [based on histological analysis, advanced cardiac imaging (cardiac MRI and/or FDG PET/CT imaging), etc.]. Finally, because the NRD is a US national database and lacks information about regional differences and patient race/ethnicity, we are unable to estimate these potential sources of differences.

## Conclusions

In a nationally representative cohort of US hospitalizations for heart failure, ventricular arrythmias, heart block, or cardiac arrest, cases with secondary diagnosis of CS (CS+) were more likely to be younger males and to present with ventricular tachycardia (VT) or heart block compared to patients without a secondary CS diagnosis (CS−). CS+ cases were more likely to undergo invasive diagnosis and therapeutic procedures, including temporary mechanical circulatory support and heart replacement therapies. Despite having higher predicted mortality risk, in-hospital mortality and 30-day readmission rates were similar to CS− cases. These findings could inform diagnostic and therapeutic approaches for clinicians confronting patients with unexplained HF or arrhythmias.

## Data Availability

The dataset presented in this article is available for purchase from the Agency for Healthcare Research and Quality (AHRQ) Healthcare Cost and Utilization Project (HCUP). Requests to access the dataset should be directed to https://hcup-us.ahrq.gov/tech_assist/centdist.jsp.

## Supplementary material

Supplementary Figure S1 Standardized Mean Difference Plot. The CS− unmatched population is shown in blue and the CS− population after propensity score matching is shown in red. The two dashed lines represent the recommended limits of −0.25 and 0.25 for the standardized mean differences.

Supplementary Figure S2 Index Event Mortality **(A)** and 30-day Readmission **(B)** Over Time. CS, cardiac sarcoidosis.

Supplementary Table S1 Additional Comorbidities. CS, cardiac sarcoidosis; APR-DRG, all patients refined diagnosis related groups. Data presented as *n* (%) unless otherwise noted.
