# Supplementary material for: Hospitalizations and cardiac sarcoidosis: insights into presentation and diagnosis from the nationwide readmission database
Source: Front Cardiovasc Med. 2024 Nov 19;11:1475181. doi: 10.3389/fcvm.2024.1475181 (PMC11612000; doi:10.3389/fcvm.2024.1475181)
Supplement: Supplementary Table S1 — Additional Comorbidities. CS, cardiac sarcoidosis; APR-DRG, all patients refined diagnosis related groups. Data presented as n (%) unless otherwise noted. [file Datasheet1.pdf]

Hospitalizations and Cardiac Sarcoidosis: Insights into Presentation and Diagnosis from the Nationwide Readmission Database

Jacob Abraham[1]*, Kateri Spinelli[1], Hsin-Fang Li[1], Tuan Pham[2], Mansen Wang[1] and Farooq H. Sheikh[3]

[1]Center for Cardiovascular Analytics, Research and Data Science (CARDS), Providence Heart Institute, Providence Research Network, Portland, Oregon, [2]Adventist Health, Portland, Oregon, [3]Advanced Heart Failure Program, MedStar Washington Hospital Center, Georgetown University School of Medicine, Washington, DC

SUPPLEMENTARY TABLES & FIGURES

Supplementary Table S1: Additional Comorbidities

Variable	CS+ (N=1,146)	CS- (N=3,250,696)
Additional comorbidities		
Acquired immune deficiency syndrome	2 (0.2%)	10667 (0.3%)
Alcohol abuse	38 (3%)	128545 (4%)
Arthropathies	42 (4%)	116146 (4%)
Lymphoma	8 (1%)	30725 (1%)
Leukemia	5 (0.4%)	22741 (1%)
Metastatic cancer	1 (0.1%)	41641 (1%)
Solid tumor without metastasis, in situ	0 (0%)	775 (0.02%)
Solid tumor without metastasis, malignant	13 (1%)	81302 (3%)
Congestive heart failure	24 (2%)	5423 (0.2%)
Dementia	4 (0.4%)	253764 (8%)
Depression	103 (9%)	343204 (11%)
Drug abuse	17 (2%)	93312 (3%)
Liver disease, moderate to severe	1 (0.1%)	3844 (0.1%)
Paralysis	10 (1%)	61213 (2%)
Hypothyroidism	151 (13%)	573905 (18%)
Other thyroid disorders	43 (4%)	57652 (2%)
Weight loss	0 (0%)	2 (0%)
APR-DRG risk of mortality		
Minor likelihood of dying	169 (15%)	565389 (17%)
Moderate likelihood of dying	336 (29%)	1043888 (32%)
Major likelihood of dying	486 (42%)	1185370 (36%)
Extreme likelihood of dying	155 (13%)	455937 (14%)
APR-DRG severity of illness		
Minor loss of function (includes cases with no comorbidity or complications)	192 (17%)	431075 (13%)
Moderate loss of function	444 (39%)	1180705 (36%)
Major loss of function	370 (32%)	1237198 (38%)
Extreme loss of function	140 (12%)	401606 (12%)

Abbreviations: CS = Cardiac sarcoidosis, APR-DRG = All Patients Refined Diagnosis Related Groups
Data presented as n (%) unless otherwise noted.

Supplementary Figure S1: Standardized Mean Difference Plot. The CS- unmatched population is shown in blue and the CS- population after propensity score matching is shown in red. The two dashed lines represent the recommended limits of -0.25 and 0.25 for the standardized mean differences.

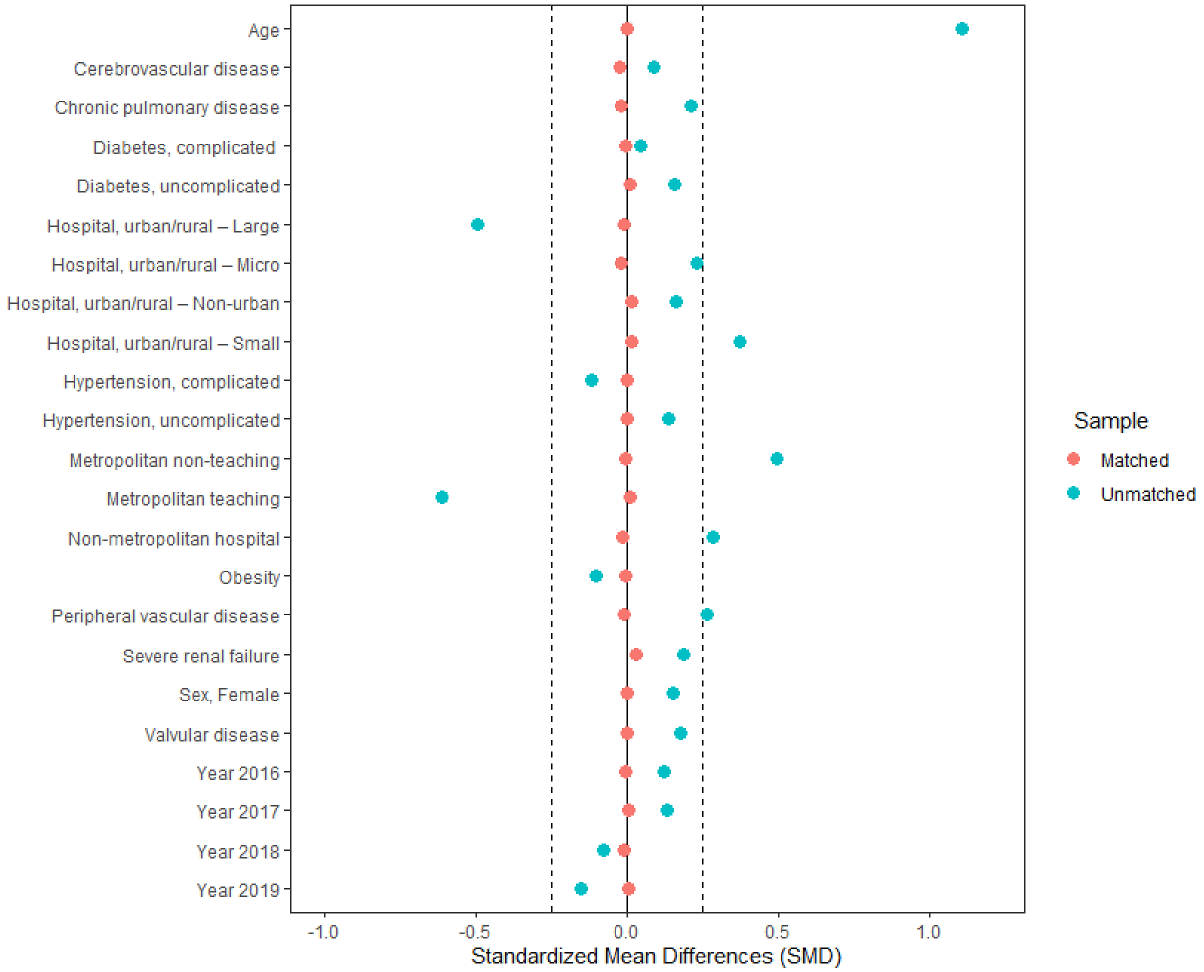

Supplementary Figure S2: Index Event Mortality (A) and 30-day Readmission (B) Over Time. CS = cardiac sarcoidosis.

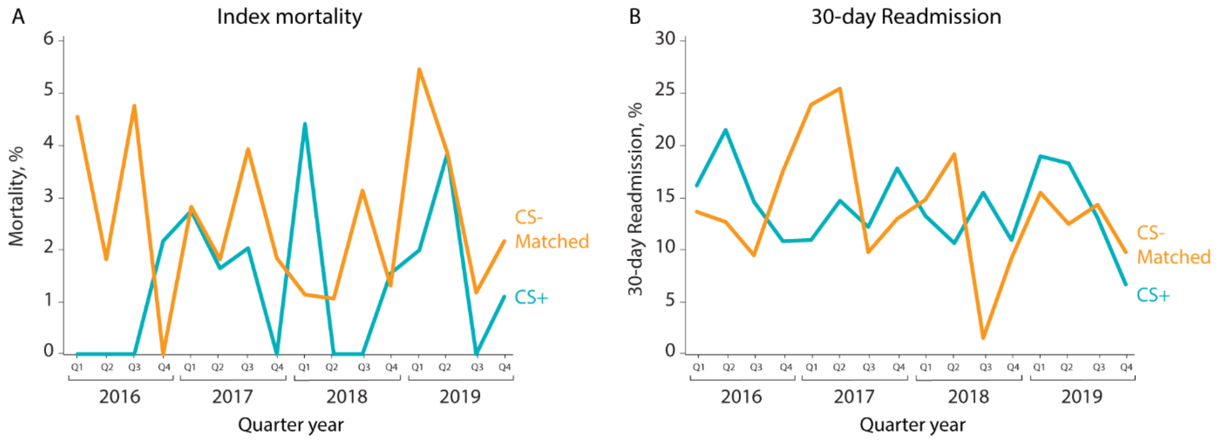

Supplementary Table S2: ICD-10 Codes

**Element and Code Description**	**Code**
Cardiac Sarcoidosis	
Sarcoid myocarditis (cardiac sarcoidosis)	D86.85
Heart Failure	
Hypertensive heart disease with heart failure	I11.0
Hypertensive heart and chronic kidney disease with heart failure and stage 1 through stage 4 chronic kidney disease, or unspecified chronic kidney disease	I13.0
Hypertensive heart and chronic kidney disease with heart failure and stage 5 chronic kidney disease, or end stage renal disease	I13.2
Heart failure	I50
Left ventricular failure, unspecified	I50.1
Unspecified systolic (congestive) heart failure	I50.20
Acute systolic (congestive) heart failure	I50.21
Chronic systolic (congestive) heart failure	I50.22
Acute on chronic systolic (congestive) heart failure	I50.23
Unspecified diastolic (congestive) heart failure	I50.30
Acute diastolic (congestive) heart failure	I50.31
Chronic diastolic (congestive) heart failure	I50.32
Acute on chronic diastolic (congestive) heart failure	I50.33
Unspecified combined systolic (congestive) and diastolic (congestive) heart failure	I50.40
Acute combined systolic (congestive) and diastolic (congestive) heart failure	I50.41
Chronic combined systolic (congestive) and diastolic (congestive) heart failure	I50.42
Acute on chronic combined systolic (congestive) and diastolic (congestive) heart failure	I50.43
Right heart failure, unspecified	I50.810
Acute right heart failure	I50.811
Chronic right heart failure	I50.812
Acute on chronic right heart failure	I50.813
Right heart failure due to left heart failure	I50.814
Biventricular heart failure	I50.82
End stage heart failure	I50.84
Other heart failure	I50.89
Heart failure, unspecified	I50.9
Cardiac Arrest	
Cardiac arrest due to an underlying cardiac condition	I46.2

Element and Code Description	Code
Cardiac arrest due to other underlying condition	I46.8
Cardiac arrest, cause unspecified	I46.9
Heart Failure/Cardiomyopathy	
Other cardiomyopathies	I42.8
Cardiomyopathy, unspecified	I42.9
Dilated cardiomyopathy	I42.0
Other hypertrophic cardiomyopathy	I42.2
Other restrictive cardiomyopathy	I42.5
Cardiomyopathy in diseases classified elsewhere	I43
General arrhythmia	
Unspecified premature depolarization	I49.40
Other premature depolarization	I49.49
Other specified cardiac arrhythmias	I49.8
Cardiac arrhythmia, unspecified	I49.9
Paroxysmal tachycardia	I47
Ventricular arrhythmia	
Ventricular tachycardia	I47.2
Paroxysmal tachycardia, unspecified	I47.9
Tachycardia, unspecified	R000
Re-entry ventricular arrhythmia	I47.0
Ventricular tachycardia	I47.2
Paroxysmal tachycardia, unspecified	I47.9
Ventricular fibrillation	I49.01
Ventricular flutter	I49.02
Ventricular premature depolarization	I49.3
Supraventricular arrhythmia	
Supraventricular tachycardia	I47.1
Paroxysmal atrial fibrillation	I48.0
Persistent atrial fibrillation	I48.1
Chronic atrial fibrillation	I48.2
Typical atrial flutter	I48.3
Atypical atrial flutter	I48.4
Unspecified atrial fibrillation	I48.91
Unspecified atrial flutter	I48.92

Element and Code Description	Code
Heart Block	
Atrioventricular block, first degree	I44.0
Atrioventricular block, second degree	I44.1
Atrioventricular block, complete	I44.2
Unspecified atrioventricular block	I44.30
Other atrioventricular block	I44.39
Left anterior fascicular block	I44.4
Left posterior fascicular block	I44.5
Unspecified fascicular block	I44.60
Other fascicular block	I44.69
Left-bundle-branch block, unspecified	I44.7
Right fascicular block	I45.0
Unspecified right bundle-branch block	I45.10
Other right bundle-branch block	I45.19
Bifascicular block	I45.2
Trifascicular block	I45.3
Nonspecific intraventricular block	I45.4
Other specified heart block	I45.5
Pacemaker Defibrillator Procedures	
Insertion of Cardiac Resynchronization Pacemaker Pulse Generator into Abdomen Subcutaneous Tissue and Fascia, Percutaneous Approach	0JH837Z
Insertion of Defibrillator Generator into Abdomen Subcutaneous Tissue and Fascia, Percutaneous Approach	0JH838Z
Insertion of Cardiac Resynchronization Defibrillator Pulse Generator into Abdomen Subcutaneous Tissue and Fascia, Percutaneous Approach	0JH839Z
Insertion of Cardiac Rhythm Related Device into Abdomen Subcutaneous Tissue and Fascia, Percutaneous Approach	0JH83PZ
Ventricular Assist Device (VAD) Procedures	
Insertion of Implantable Heart Assist System into Heart, Open Approach	02HA0QZ
Insertion of Implantable Heart Assist System into Heart, Percutaneous Approach	02HA3QZ
Insertion of Implantable Heart Assist System into Heart, Percutaneous Endoscopic Approach	02HA4QZ
Assistance with Cardiac Output using Pulsatile Compression, Intermittent	5A02115
Assistance with Cardiac Output using Other Pump, Intermittent	5A02116
Assistance with Cardiac Output using Impeller Pump, Intermittent	5A0211D

Element and Code Description	Code
Assistance with Cardiac Output using Pulsatile Compression, Continuous	5A02215
Assistance with Cardiac Output using Other Pump, Continuous	5A02216
Assistance with Cardiac Output using Impeller Pump, Continuous	5A0221D
Temporary Mechanical Cardiac Support (tMCS) Procedures	
Insertion of Short-term External Heart Assist System into Heart, Intraoperative, Open Approach	02HA0RJ
Insertion of Biventricular Short-term External Heart Assist System into Heart, Open Approach	02HA0RS
Insertion of Short-term External Heart Assist System into Heart, Open Approach	02HA0RZ
Insertion of Short-term External Heart Assist System into Heart, Intraoperative, Percutaneous Approach	02HA3RJ
Insertion of Biventricular Short-term External Heart Assist System into Heart, Percutaneous Approach	02HA3RS
Insertion of Short-term External Heart Assist System into Heart, Percutaneous Approach	02HA3RZ
Insertion of Short-term External Heart Assist System into Heart, Intraoperative, Percutaneous Endoscopic Approach	02HA4RJ
Insertion of Biventricular Short-term External Heart Assist System into Heart, Percutaneous Endoscopic Approach	02HA4RS
Insertion of Short-term External Heart Assist System into Heart, Percutaneous Endoscopic Approach	02HA4RZ
Assistance with Cardiac Output using Balloon Pump, Intermittent	5A02110
Assistance with Cardiac Output using Balloon Pump, Continuous	5A0221D
Extracorporeal Membrane Oxygenation (ECMO) Procedures	
Extracorporeal Supersaturated Oxygenation, Intermittent	5A0512C
Extracorporeal Supersaturated Oxygenation, Continuous	5A0522C
Extracorporeal Membrane Oxygenation, Continuous	5A15223
Extracorporeal Oxygenation, Membrane, Central	5A1522F
Extracorporeal Oxygenation, Membrane, Peripheral Veno-arterial	5A1522G
Extracorporeal Oxygenation, Membrane, Peripheral Veno-venous	5A1522H
Transplant Procedures	
Transplantation of Heart, Allogeneic, Open Approach	02YA0Z0
Fluorine 18 Positron Emission Tomography	
Positron Emission Tomographic (PET) Imaging of Myocardium using Fluorine 18	C23GK
Positron Emission Tomographic (PET) Imaging of Myocardium using Fluorine 18	C23GKZ
Positron Emission Tomographic (PET) Imaging of Myocardium using Fluorine 18	C23GKZZ

Element and Code Description	Code
Cardiac Magnetic Resonance Imaging	
Magnetic Resonance Imaging (MRI) of Right and Left Heart	B236
Magnetic Resonance Imaging (MRI) of Right and Left Heart, Other Contrast	B236Y
Magnetic Resonance Imaging (MRI) of Right and Left Heart, Other Contrast, Unenhanced and Enhanced	B236Y0
Magnetic Resonance Imaging (MRI) of Right and Left Heart using Other Contrast, Unenhanced and Enhanced	B236Y0Z
Magnetic Resonance Imaging (MRI) of Right and Left Heart using Other Contrast	B236YZ
Magnetic Resonance Imaging (MRI) of Right and Left Heart using Other Contrast	B236YZZ
Magnetic Resonance Imaging (MRI) of Right and Left Heart	B236Z
Magnetic Resonance Imaging (MRI) of Right and Left Heart	B236ZZ
Magnetic Resonance Imaging (MRI) of Right and Left Heart	B236ZZZ
Right Heart Catheterization (RHC) Procedures	
Measurement of Cardiac Sampling and Pressure, Right Heart, Open Approach	4A020N6
Measurement of Cardiac Sampling and Pressure, Bilateral, Open Approach	4A020N8
Measurement of Cardiac Sampling and Pressure, Right Heart, Percutaneous Approach	4A023N6
Measurement of Cardiac Sampling and Pressure, Bilateral, Percutaneous Approach	4A023N8
Measurement of Cardiac Sampling and Pressure, Right Heart, Via Natural or Artificial Opening	4A027N6
Measurement of Cardiac Sampling and Pressure, Bilateral, Via Natural or Artificial Opening	4A027N8
Measurement of Cardiac Sampling and Pressure, Right Heart, Via Natural or Artificial Opening Endoscopic	4A028N6
Measurement of Cardiac Sampling and Pressure, Bilateral, Via Natural or Artificial Opening Endoscopic	4A028N8
Coronary Angiography Procedures	
Plain Radiography of Single Coronary Artery using High Osmolar Contrast	B2000ZZ
Plain Radiography of Single Coronary Artery using Low Osmolar Contrast	B2001ZZ
Plain Radiography of Single Coronary Artery using Other Contrast	B200YZZ
Plain Radiography of Multiple Coronary Arteries using High Osmolar Contrast	B2010ZZ
Plain Radiography of Multiple Coronary Arteries using Low Osmolar Contrast	B2011ZZ
Plain Radiography of Multiple Coronary Arteries using Other Contrast	B201YZZ
Plain Radiography of Single Coronary Artery Bypass Graft using High Osmolar Contrast	B2020ZZ
Plain Radiography of Single Coronary Artery Bypass Graft using Low Osmolar Contrast	B2021ZZ

Element and Code Description	Code
Plain Radiography of Single Coronary Artery Bypass Graft using Other Contrast	B202YZZ
Plain Radiography of Multiple Coronary Artery Bypass Grafts using High Osmolar Contrast	B2030ZZ
Plain Radiography of Multiple Coronary Artery Bypass Grafts using Low Osmolar Contrast	B2031ZZ
Plain Radiography of Multiple Coronary Artery Bypass Grafts using Other Contrast	B203YZZ
Plain Radiography of Right Internal Mammary Bypass Graft using High Osmolar Contrast	B2070ZZ
Plain Radiography of Right Internal Mammary Bypass Graft using Low Osmolar Contrast	B2071ZZ
Plain Radiography of Right Internal Mammary Bypass Graft using Other Contrast	B207YZZ
Plain Radiography of Left Internal Mammary Bypass Graft using High Osmolar Contrast	B2080ZZ
Plain Radiography of Left Internal Mammary Bypass Graft using Low Osmolar Contrast	B2081ZZ
Plain Radiography of Left Internal Mammary Bypass Graft using Other Contrast	B208YZZ
Plain Radiography of Other Bypass Graft using High Osmolar Contrast	B20F0ZZ
Plain Radiography of Other Bypass Graft using Low Osmolar Contrast	B20F1ZZ
Plain Radiography of Other Bypass Graft using Other Contrast	B20FYZZ
Fluoroscopy of Single Coronary Artery using High Osmolar Contrast, Laser Intraoperative	B210010
Fluoroscopy of Single Coronary Artery using High Osmolar Contrast	B2100ZZ
Fluoroscopy of Single Coronary Artery using Low Osmolar Contrast, Laser Intraoperative	B210110
Fluoroscopy of Single Coronary Artery using Low Osmolar Contrast	B2101ZZ
Fluoroscopy of Single Coronary Artery using Other Contrast, Laser Intraoperative	B210Y10
Fluoroscopy of Single Coronary Artery using Other Contrast	B210YZZ
Fluoroscopy of Multiple Coronary Arteries using High Osmolar Contrast, Laser Intraoperative	B211010
Fluoroscopy of Multiple Coronary Arteries using High Osmolar Contrast	B2110ZZ
Fluoroscopy of Multiple Coronary Arteries using Low Osmolar Contrast, Laser Intraoperative	B211110
Fluoroscopy of Multiple Coronary Arteries using Low Osmolar Contrast	B2111ZZ
Fluoroscopy of Multiple Coronary Arteries using Other Contrast, Laser Intraoperative	B211Y10
Fluoroscopy of Multiple Coronary Arteries using Other Contrast	B211YZZ
Fluoroscopy of Single Coronary Artery Bypass Graft using High Osmolar Contrast	B2120ZZ

Element and Code Description	Code
Fluoroscopy of Single Coronary Artery Bypass Graft using Low Osmolar Contrast, Laser Intraoperative	B212110
Fluoroscopy of Single Coronary Artery Bypass Graft using Low Osmolar Contrast	B2121ZZ
Fluoroscopy of Single Coronary Artery Bypass Graft using Other Contrast, Laser Intraoperative	B212Y10
Fluoroscopy of Single Coronary Artery Bypass Graft using Other Contrast	B212YZZ
Fluoroscopy of Multiple Coronary Artery Bypass Grafts using High Osmolar Contrast, Laser Intraoperative	B213010
Fluoroscopy of Multiple Coronary Artery Bypass Grafts using High Osmolar Contrast	B2130ZZ
Fluoroscopy of Multiple Coronary Artery Bypass Grafts using Low Osmolar Contrast, Laser Intraoperative	B213110
Fluoroscopy of Multiple Coronary Artery Bypass Grafts using Low Osmolar Contrast	B2131ZZ
Fluoroscopy of Multiple Coronary Artery Bypass Grafts using Other Contrast, Laser Intraoperative	B213Y10
Fluoroscopy of Multiple Coronary Artery Bypass Grafts using Other Contrast	B213YZZ
Fluoroscopy of Right Internal Mammary Bypass Graft Using High Osmolar Contrast	B2170ZZ
Fluoroscopy of Right Internal Mammary Bypass Graft Using Low Osmolar Contrast	B2171ZZ
Fluoroscopy of Right Internal Mammary Bypass Graft Using Other Contrast	B217YZZ
Fluoroscopy of Left Internal Mammary Bypass Graft Using High Osmolar Contrast	B2180ZZ
Fluoroscopy of Left Internal Mammary Bypass Graft Using Low Osmolar Contrast	B2181ZZ
Fluoroscopy of Left Internal Mammary Bypass Graft Using Other Contrast	B218YZZ
Fluoroscopy of Other Bypass Graft using High Osmolar Contrast	B21F0ZZ
Fluoroscopy of Other Bypass Graft using Low Osmolar Contrast	B21F1ZZ
Fluoroscopy of Other Bypass Graft using Other Contrast	B21FYZZ
Endomyocardial Biopsy Procedures	
Excision of Right Ventricle, Open Approach, Diagnostic	02BK0ZX
Excision of Right Ventricle, Percutaneous Approach, Diagnostic	02BK3ZX
Excision of Right Ventricle, Perc Endo Approach, Diagnostic	02BK4ZX
Excision of Ventricular Septum, Open Approach, Diagnostic	02BM0ZX
Excision of Ventricular Septum, Percutaneous Approach, Diagnostic	02BM3ZX
Excision of Ventricular Septum, Perc Endo Approach, Diagnostic	02BM4ZX
